# Cardiac Tamponade from Chylopericardium Following Lobectomy and Mediastinal Lymph Node Dissection for Lung Cancer: A Case Report

**DOI:** 10.70352/scrj.cr.25-0365

**Published:** 2025-11-29

**Authors:** Masakazu Katsura, Masayoshi Umesue, Naoya Otaka, Masayuki Shimada, Noriko Fujimoto, Tsukihisa Yoshida, Takeshi Matsuda, Keiichi Kikuchi, Mitsuhiro Takenoyama

**Affiliations:** 1 Department of Thoracic Surgery, Red Cross Matsuyama Hospital, Matsuyama, Ehime, Japan; 2 Department of Cardiovascular Surgery, Red Cross Matsuyama Hospital, Matsuyama, Ehime, Japan; 3 Department of Cardiology, Red Cross Matsuyama Hospital, Matsuyama, Ehime, Japan; 4 Department of Diagnostic and Therapeutic Radiology, Red Cross Matsuyama Hospital, Matsuyama, Ehime, Japan

**Keywords:** cardiac tamponade, chylopericardium, lobectomy, lymph node dissection, lung cancer

## Abstract

**INTRODUCTION:**

Chylopericardium is a rare but serious complication after thoracic surgery, caused by injury to thoracic duct tributaries during procedures like lymph node dissection. It leads to chylous fluid in the pericardial cavity. Prompt diagnosis is vital to prevent cardiac tamponade, malnutrition, and immunosuppression due to disrupted lymphatic drainage.

**CASE PRESENTATION:**

A 74-year-old man presented with neck discomfort, and a CT scan incidentally revealed a part-solid ground-glass nodule (GGN) in the right upper lung lobe, raising suspicion of primary lung cancer. Thoracoscopic lobectomy was performed, followed by mediastinal dissection, during which a small pericardial perforation occurred. On POD 4, the patient developed sudden obstructive shock secondary to cardiac tamponade. After consultation with the cardiology, cardiac surgery, and radiology departments, aortic dissection was initially suspected as the most likely cause. As a result of exploratory thoracotomy via cardiac surgery, no aortic dissection was found. Chylous fluid was found in the pericardial space and was thought to be the cause of cardiac tamponade. Conservative management of the chylous effusion was unsuccessful, and thoracoscopic ligation of the lymphatic vessels was required. No reaccumulation of the chylous pericardial fluid was observed after the intervention.

**CONCLUSIONS:**

Following lung surgery, particularly when lymph node dissection or unintentional pericardial breach is performed, chylous pericardial effusion accompanied by cardiac tamponade may occur. Therefore, this complication should be considered.

## Abbreviations

CTR
: cardiothoracic ratio
GGN
: ground-glass nodule

## INTRODUCTION

Chylopericardium represents a rare but potentially life-threatening complication following thoracic surgery, characterized by the accumulation of chylous fluid within the pericardial cavity.^1)^ This condition occurs in 0.22%–0.5% of patients following intrathoracic procedures, with iatrogenic injury to thoracic duct tributaries during mediastinal lymph node dissection being a recognized etiology.^2)^ The pathophysiology involves disruption of lymphatic drainage pathways, leading to abnormal communication between damaged lymphatic vessels and the pericardial space. Early recognition and appropriate management are crucial, as delayed diagnosis can result in cardiac tamponade, nutritional depletion, and immunosuppression.^2)^ This case report presents a 74-year-old patient who developed chylopericardium following thoracoscopic right upper lobectomy with superior mediastinal dissection, highlighting the diagnostic challenges and therapeutic strategies for this uncommon postoperative complication.

## CASE PRESENTATION

**Fig. 1:**
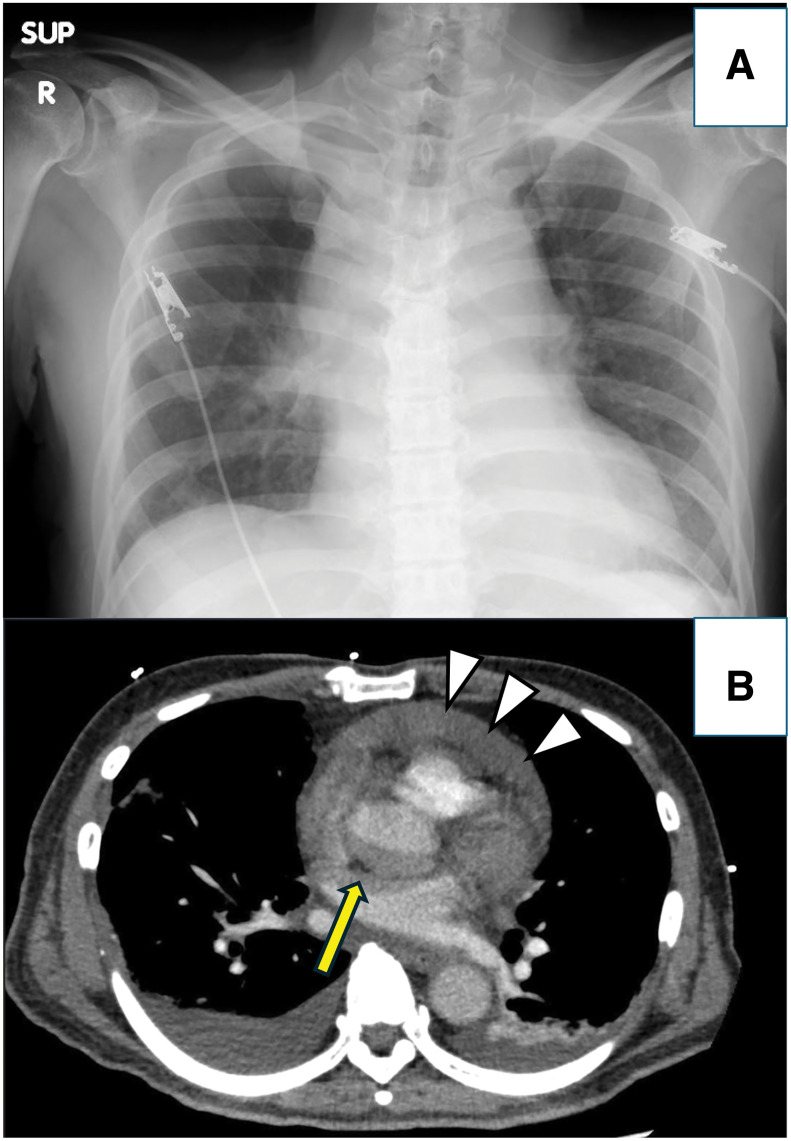
(**A**) A chest radiograph taken immediately after the onset of shock suggested a slight enlargement of the cardiac silhouette. The calculated CTR was 58%. (**B**) CT revealed evidence of pericardial effusion. Contrast density indicates blood (arrowheads). Aortic dissection was suspected based on the imaging findings (yellow arrow). CTR, cardiothoracic ratio

**Fig. 2:**
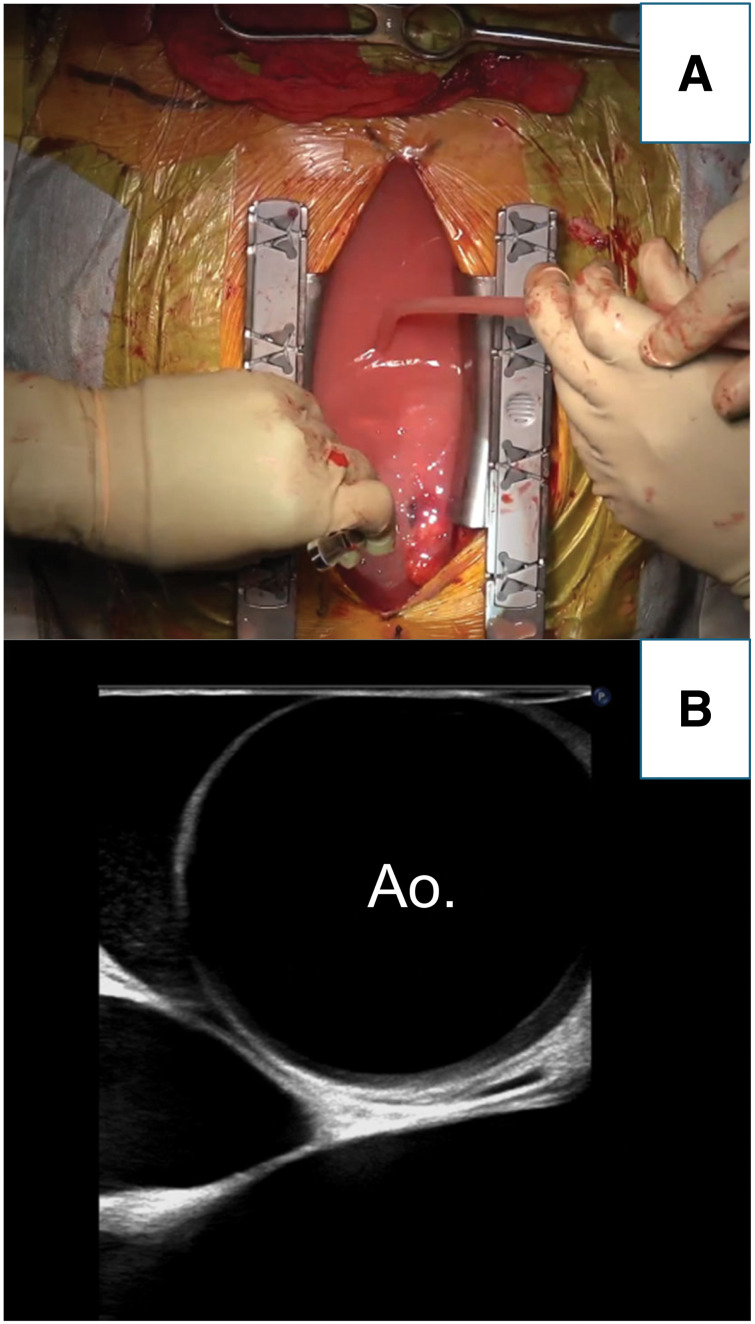
(**A**) Chylous pericardial effusion drained via pericardiotomy. (**B**) The echocardiogram performed during surgery did not reveal any signs of aortic dissection (ascending aorta: Ao.).

**Fig. 3:**
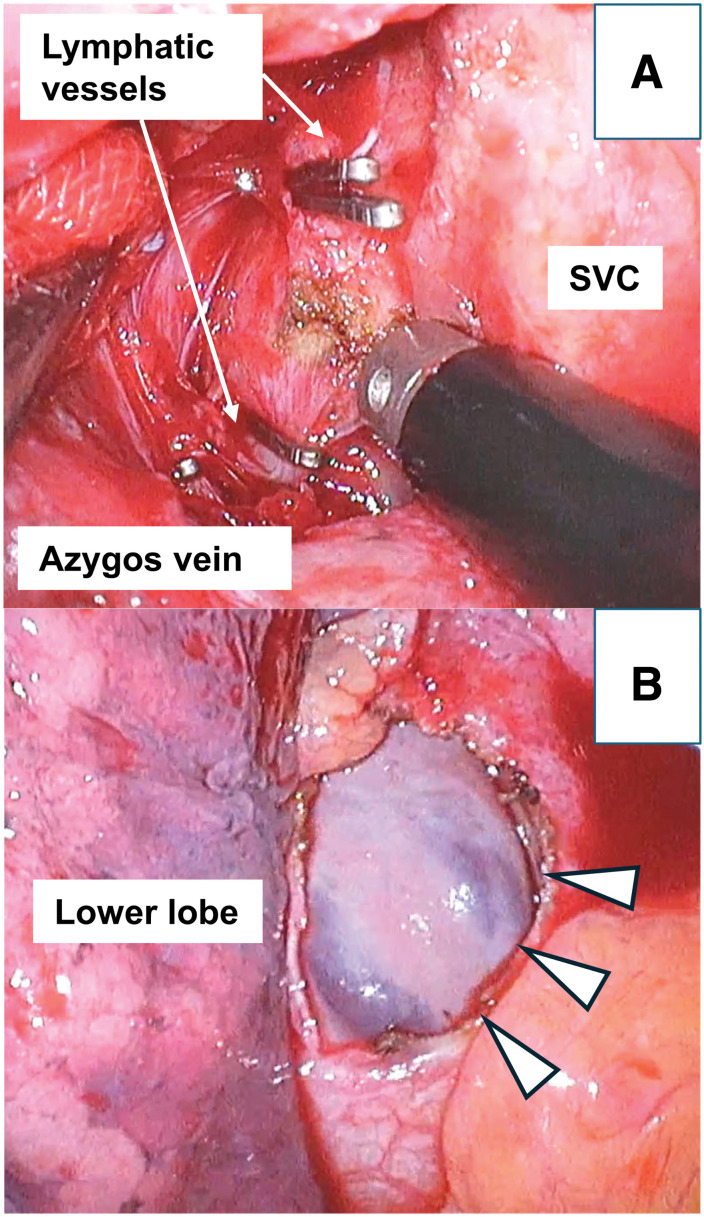
(**A**) The lymphatic vessels were ligated using clips. We confirmed that the discharge of the chyle stopped. (**B**) Pericardial fenestration was then performed.

**Fig. 4:**
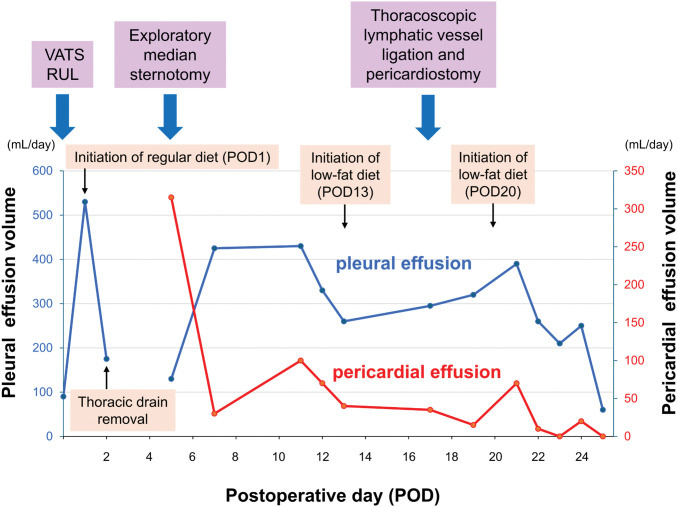
The *x*-axis shows POD. The left *y*-axis indicates daily pleural effusion volume (mL/day), and the right *y*-axis indicates daily pericardial effusion volume (mL/day). Volumes represent 24-hour drainage outputs. The timing of key events is annotated on the plot: initiation of a regular diet (POD 1), initiation/re-initiation of a low-fat diet (POD 13 and POD 20), thoracic drain removal, and surgical procedures (VATS right upper lobectomy [RUL], exploratory median sternotomy, and thoracoscopic lymphatic vessel ligation with pericardiostomy). RUL, right upper lobectomy; VATS, video-assisted thoracoscopic surgery

A 74-year-old man visited his family doctor due to neck discomfort, and CT incidentally revealed a part-solid ground-glass nodule (GGN) in the upper lobe of the right lung (segment 1, S1). Thoracoscopic right upper lobe resection with lymph node dissection was performed for suspected primary lung cancer. We usually perform thoracoscopic surgery using a multiportal approach with four ports. There was no pleural dissemination or adhesion to the thoracic cavity. The right upper lobe of the lung was resected without any complications. Subsequently, superior mediastinal dissection was performed. The small hole was created while dissecting the pericardial side of the aortic surface during mediastinal lymph node dissection. The lymphatic vessels were closed using an energy device or clip, as appropriate. We applied fibrin glue to the staple line and the dissection site of the mediastinal lymph node. The purpose was to reinforce the staple line and prevent minor chyle leakage and oozing from dissection site of the mediastinal lymph node. No special treatment was performed on this small hole. The patient’s food intake was good after POD 1. On POD 2, the amount of pleural fluid was 175 mL and its nature was serous. The chest drain was then removed on the same day. The patient had consumed his breakfast before the removal. The patient’s condition was good until the 4th POD. Chest radiography revealed a normal cardiothoracic ratio (CTR) and no obvious abnormalities. On POD 4 at night, the patient experienced chest discomfort and severe hypotension. Based on the echocardiogram and contrast CT findings, the cardiologist suspected that the patient had obstructive shock and cardiac tamponade due to aortic dissection (**Fig. 1**). Emergency surgery was performed at the cardiac surgery department. When the pericardium was opened experimentally via a median sternotomy, chylous pericardial fluid was observed. Based on the intraoperative echo findings, no findings of aortic dissection were observed (**Fig. 2**). Chest and pericardial drainage, fasting, central venous nutrition, and coagulation factor (factor XIII) were administered, but no improvement in pleural effusion volume was observed. On the 17th day after lung resection, thoracoscopic lymphatic vessel ligation and pericardiostomy were performed. The space created after the upper mediastinal dissection was completely occupied by the remaining middle lobe, suggesting that chylous fluid had flowed into the pericardium rather than the thoracic cavity. The middle lobe was adhered to the upper mediastinum, making the identification of the chyle leakage site difficult. Therefore, we carefully removed the adhesion. During surgery, milk was administered via a feeding tube to identify the lymphatic leakage site. The leakage stopped when multiple thin lymphatic vessels suspected of leaking were clipped. The middle lobe was adhered to the upper mediastinum, making identification of the chyle leakage site difficult. Therefore, we carefully removed the adhesion. The thoracic duct was not ligated. Our priority was to identify the site of chyle leakage and ligate the leaking lymphatic vessels. If this proved impossible, we planned to ligate the thoracic duct and made preparations accordingly. Two anterior pericardiectomies were performed in front of the lower inferior vena cava, with resection sizes of 2 × 3 cm and 2 × 2 cm, respectively (**Fig. 3**). The relationship between time-course changes in pleural and pericardial effusion volume and surgery is shown in **Fig. 4**. Three years have passed since the surgery with no recurrence of chylous pericardial effusion.

## DISCUSSION

Chylous pericardial effusion is a rare complication that occurs after thoracic surgery, with an incidence ranging from 0.2% to 0.5%.^3)^ The incidence of chylous pericardial effusion combined with chylothorax is low.^4)^ Chylous pericarditis is a disorder characterized by the accumulation of chylous fluid in the pericardial cavity resulting from multiple etiologies.^5)^ Primary chylous pericarditis is defined as having no discernible cause or resulting from lymphatic malformations,^6)^ whereas secondary cases are attributed to identifiable factors such as trauma or thoracic surgery. Most secondary chylous pericarditis cases are documented as complications following cardiac surgery, with only 12 instances reported after non-cardiac procedures such as lung cancer surgery, including the present case, based on our research findings.^7)^ Moreover, instances of diagnosis induced by cardiac tamponade, as exemplified in this case, are infrequent, occurring in approximately 4 of 12 cases.^3,7,8)^ No definitive idea exists about the process underlying chylothorax development after lung cancer surgery; however, it is generally believed to result from disrupted lymphatic drainage into the pericardial cavity or thoracic duct system due to lymph node dissection. Thoracoscopic surgery provides enhanced visualization of vascular and lymphatic structures due to magnification, making it more suitable for precise dissection and management. In this case, thoracoscopic surgery facilitated accurate lymphatic ligation and pericardiectomy.

Riquet et al. documented the lymphatic pathways in the pericardial cavity across 90 autopsy cases, discovering that most lymph from the left ventricle is sent through the lower right paratracheal lymph nodes.^9)^ Meanwhile, the causes of cardiac tamponade after lung cancer surgery can be broadly divided into chylothorax and intrapericardial bleeding.^10)^ In this case, the following mechanisms are presumed. The pericardium was incised during the lymph node dissection. During this series of surgical procedures, the possibility of direct damage to intrapericardial lymphatic vessels was minimal. The lymphatic channel that exhibits a breach around the upper mediastinal lymph node flows directly into the pericardium. The small hole in the pericardium was accidentally covered by the middle lobe, and the adhesion was further strengthened by fibrin glue. This mechanism causes the accumulation of chylous fluid in the pericardium. It was not evacuated by the thoracic drain into the thoracic cavity, resulting in cardiac tamponade. Although minor pericardial perforations during upper mediastinal lymph node dissection are often managed conservatively, inadvertent sealing with fibrin glue may rarely lead to chylopericardium. This highlights the need for careful intraoperative decision-making and vigilant postoperative surveillance. Following this case, we made several specific changes. Only when the pericardium of the superior mediastinum is injured, we discontinued the application of fibrin glue to the superior mediastinum for prophylactic sealing. We now make every effort to clip any lymphatic ducts exposed during dissection. Postoperatively, we carefully monitor for changes in drain output characteristics, vital signs, cardiac shadow on plain chest X-ray, and chest symptoms after oral intake and promptly consult the cardiology team in case of sudden changes.

The reason why the patient’s chylous pericardial effusion appeared on contrast-enhanced CT to mimic a type A aortic dissection requires clarification. As a result, the gray, crescent-like shadow of the ascending aorta indicated by the yellow arrow in **Fig. 1B** represents chylous pericardial effusion. The superior pericardial recess, particularly its posterior portion, may appear as a low-attenuation crescent-shaped shadow behind the ascending aorta.^11,12)^ Because chylous effusion has lower HU values than hemopericardium or hydropericardium,^13)^ it can resemble an intimal flap, false lumen, or mediastinal mass. Recognition of these anatomical variations and imaging characteristics is crucial, and ECG-gated thin-section CT with multiplanar reformation would have improved diagnostic accuracy.^14)^

Pericardial drainage was considered before surgery; however, we hesitated to perform drainage due to the risk of promoting bleeding and worsening hemodynamic instability because acute type A dissection with rupture was strongly suspected. Before surgery, the radiologist, cardiologist, and cardiac surgeons, including us, all judged the crescent-shaped shadow to be a dissection cavity, not a chylous pericardial effusion. Ultimately, we proceeded to median sternotomy. Postoperatively, we reviewed the case with cardiologists. On retrospective evaluation, both the CT and echocardiographic findings were still interpreted as highly suspicious for aortic dissection, supporting the diagnostic challenge in this case.

## CONCLUSIONS

The present case highlights the importance of a high index of suspicion for chylous pericardial effusion in patients who underwent extensive lymph node dissection during lung cancer surgery. When pericardial manipulation is necessary, the method and timing of closure should be carefully considered, particularly regarding the use of sealing agents such as fibrin glue. Regular postoperative monitoring and early intervention are essential for optimal patient outcome. Further research is needed to establish standardized guidelines for the prevention and management of this rare but potentially life-threatening complication.

## DECLARATIONS

### Availability of data and materials

The datasets supporting the conclusions of this article are included within the article.
